# Synthesis,* In Vivo* Anti-Inflammatory Activity, and Molecular Docking Studies of New Isatin Derivatives

**DOI:** 10.1155/2016/2181027

**Published:** 2016-02-14

**Authors:** Ravi Jarapula, Kiran Gangarapu, Sarangapani Manda, Sriram Rekulapally

**Affiliations:** ^1^Department of Pharmaceutical Chemistry, University College of Pharmaceutical Sciences, Kakatiya University, Telangana 506009, India; ^2^Department of Pharmaceutical Chemistry, Chaitanya Institute of Pharmaceutical Sciences, Rampur, Warangal, Telangana 506147, India

## Abstract

A novel synthesis of 2-hydroxy-N′-(2-oxoindolin-3-ylidene) benzohydrazide derivatives was synthesized by the condensation of 2-hydroxybenzohydrazide with substituted isatins. The synthesized compounds were characterized by FT-IR, ^1^H-NMR, and mass spectral data. Further, the compounds were screened for* in vivo* anti-inflammatory activity by carrageenan induced paw edema method. The tested compounds have shown mild-to-moderate anti-inflammatory activity. The compounds** VIIc** and** VIId** exhibited 65% and 63% of paw edema reduction, respectively. The molecular docking studies were also carried out into the active site of COX-1 and COX-2 enzymes (PDB ID: 3N8Y, 3LN1, resp.) using VLife MDS 4.3. The compounds** VIIc**,** VIId**, and** VIIf** exhibited good docking scores of −57.27, −62.02, and −58.18 onto the active site of COX-2 and least dock scores of −8.03, −9.17, and −8.94 on COX-1 enzymes and were comparable with standard COX-2 inhibitor celecoxib. A significant correlation was observed between the* in silico* and the* in vivo* studies. The anti-inflammatory and docking results highlight the fact that the synthesized compounds** VIIc**,** VIId**, and** VIIf** could be considered as possible hit as therapeutic agents.

## 1. Introduction

Cyclooxygenases (COX) or prostaglandin endoperoxide synthases are the key enzymes in the synthesis of prostaglandins, the main mediators of inflammation, pain, and increased body temperature (hyperpyrexia). The body produces two main isoforms of COX proteins, that is, cyclooxygenases-1 (COX-1) and cyclooxygenases-2 (COX-2). The COX-1 is responsible for formation of important biological mediators such as prostanoids, including prostaglandins, prostacyclin, and thromboxane, and involved in pain causing, blood clotting, and protecting the stomach [1], whereas COX-2 is involved in the pain by inflammation and plays a major role in prostaglandin biosynthesis in inflammatory cells and central nervous system [2]. When COX-1 is inhibited, inflammation is reduced, but the protection of the lining of the stomach is also lost. This can cause stomach upset as well as ulceration and bleeding from the stomach and even the intestines. Whereas COX-2 is usually specific to inflamed tissue, there is much less gastric irritation associated with COX-2 inhibition together with the decreased risk of peptic ulceration [3]. Therefore, selective COX-2 inhibitors such as celecoxib and rofecoxib had been developed for ease of inflammation associated with COX [4]. The use of coxib drugs such as rofecoxib (Vioxx^W^) and valdecoxib (Bextra^W^) was withdrawn from the market in 2004 and 2005, respectively, because of increased risk of heart attacks and strokes with long-term use [5]. At present, celecoxib (Celebrex^W^) is the only COX-2 inhibitor available in the United States. Hence, there is a need for COX-2 inhibitor with no adverse effects.

The isatin (indole-2,3-dione) pharmacophore has attracted and still attracts much attention from medicinal chemists because of great importance in their biological as well as synthetic approach of medicinal chemistry. The various derivatives of isatin are known to possess a range of biological properties including anticancer [6], anticonvulsant [7], antidepressants [8], antimicrobial [9], antiviral [10], anxiolytics activities [11], molecular docking studies of isatin derivatives as EGFR inhibitors [12], and anti-inflammatory [13], and the potent anti-inflammatory effect of indomethacin, etodolac, and tenidap encouraged us to further explore the pharmacological properties of the indole nucleus.

It has been reported that the nature of substituents at the 2- or 3-position of the indole nucleus plays an important role in modulating their anti-inflammatory properties [14–18]. Amide-containing compounds have been shown to possess a wide range of biological activities, including anti-inflammatory properties. Interestingly, the replacement of the carboxylic groups by amide groups in NSAID drugs indomethacin, meclofenamic acid, and ketoprofen conferred the compounds greater selectivity for COX-2 over the COX-1 enzyme [19].

Literature survey reveals that 4-hydroxybenzohydrazide ring is important for antimycobacterial activity [20]. In addition, many thiazoline derivatives exhibit a wide variety of biological activities such as antimicrobial [21], anti-inflammatory [22], antihistaminic [23], antihypertensive [24], hypnotic [25], and anticonvulsant [26].

**Scheme 2:**
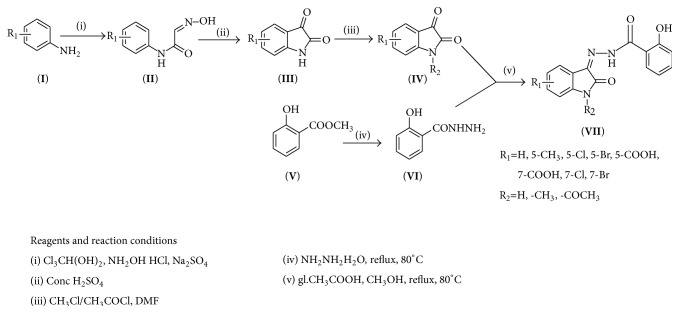
Schematic steps of 2-hydroxy-N^1^-(2-oxo-1,2-dihydro-3H-indol-3-ylidene)benzohydrazides.

Keeping in view of biological importance of the two molecular moieties, namely, isatins and 2-hydroxybenzohydrazide, to study the condensation of isatins with 2-hydroxybenzohydrazide (**II**) has been felt worthwhile as depicted in Scheme 2. The synthesized compounds were screened for* in vivo* anti-inflammatory activity and molecular docking studies. These results were supported to design novel specific inhibitors of COX-2 by the comparative modelling of COX-1 and COX-2 enzymes with the available pharmacophore. We hope that these molecules may be further explored as powerful and novel nonulcerogenic anti-inflammatory with selective inhibition of COX-2.

## 2. Materials and Methods

### 2.1. General

All the chemicals used were of analytical grade and obtained from Himedia and SD Fine Chemicals. The completion of the reaction, the purity of the compounds checked by TLC using E-Merck 0.25 mm silica gel plates, and visualization were accomplished with UV light. Melting points were determined on Bio Technics India melting point apparatus and were uncorrected. ^1^H NMR spectra were recorded on an Avance-300 MHz in CDCl_3_ solvent using TMS as an internal standard (chemical shifts in *δ*, ppm). The IR spectra were recorded on Rkin Elmer Spectrum BX-1 spectrometer by using KBr disc. The mass spectra were recorded on a LC-MSD-Trap-SL.

### 2.2. Animals

The animals were procured from Mahaveer Enterprises, Hyderabad, India. Male adult albino Wister rats (150–350 g) were acclimatized for a period of 14 days prior to performing the experiments and maintained under standard husbandry conditions and had free access to food and water* ad libitum*. The animals were divided into different groups; each consists of six animals which were fasted overnight prior to the study. The protocol of the present study was reviewed and approved by the Institutional Animal Ethical Committee.

## 3. Chemistry

### 3.1. Synthesis of Indole-2,3-diones (Isatins,** III**)

#### 3.1.1. Isonitrosoacetanilides (**I1**)

In a 5 lit. RB flask, chloral hydrate (0.54 mol) and 1200 mL of water were placed. To this solution, crystallized sodium sulphate (1300 g) was then added followed by a solution of an appropriate aromatic amine (**I**) in 300 mL of water and concentrated hydrochloric acid (0.52 mol). Finally, a solution of hydroxylamine HCl (1.58 mol) in 500 mL of water was added. The contents of the flask were heated over a wire-gauge by a Mecker burner so that vigorous boiling begins in about 45 minutes. After 1 to 2 minutes of vigorous boiling the reaction was completed. During the heating period itself the crystals of isonitrosoacetanilides started separating out. On cooling under the current of water, the entire product was solidified. It was filtered under suction, air dried, and purified by recrystallization from suitable solvent(s).

#### 3.1.2. Indole-2,3-diones (**III**)

Sulphuric acid (600 g, d, 1.84, 326 mL) were warmed at 50°C in a one liter RB flask fitted with an efficient mechanical stirrer and to this, finely powdered appropriate isonitrosoacetanilide (**II**, 0.46 mol) was added at such a rate so as to maintain the temperature between 60°C and 70°C but not higher. External cooling was applied at this stage so that the reaction could be carried out more rapidly. After the addition of isonitroso compound was completed the temperature of the solution was raised to 80°C and maintained at that temperature for 10 minutes, to complete the reaction. Then the reaction mixture was cooled to room temperature and poured on crushed ice (2.5 kg) while stirring. After standing for about half-an-hour, the product separated was filtered, washed several times with small portions of cold water, and dried. Purification of the compound was affected by the recrystallization from methanol [27].

### 3.2. Preparation of 2-Hydroxybenzohydrazide (**VI**)

In a 500 mL of RB flask, 10 g of methyl salicylate (**V**) and 50 mL of distilled alcohol were placed and the reaction mixture was shaken for 5 minutes. To this add 20 mL of hydrazine hydrate (99%) and the contents of the flask were refluxed for 3 hours, the completion of the reaction monitored by TLC. The resultant white crystalline solid was filtered and washed repeatedly, with small portions of cold alcohol. The product was dried and purified by recrystallization from methanol, yield 90%, m.p. 251–254°C [28].

### 3.3. Synthesis of 2-Hydroxy-N′-(2-oxoindolin-3-ylidene)benzohydrazide (**VII**)

A mixture of an appropriate indole-2,3-dione (0.01 mol) and 2-hydroxybenzohydrazide (0.01 mol) was taken into methanol (50 mL) in presence of glacial acetic acid which was heated under reflux on water bath for 6-7 hours. The coloured compounds were thus obtained upon cooling, were filtered, were washed with small portions of cold methanol, and were dried. They were purified by recrystallization from alcohol.

*Spectral Data of the Synthesized Compounds*

*(1) 2-Hydroxybenzohydrazide (* ***VI*** *).* IR (KBr) cm^−1^: 3320.78 (NH), 1623 (C=O), 1479 (C-N), 830–500 (Ar); MS: *m*/*z* 152 (M^+^).

*(2) 2-Hydroxy-N*′*-(2-oxoindolin-3-ylidene)benzohydrazide (* ***VIIa*** *).* IR (KBr) cm^−1^: 3169 (NH), 1735.97 (C=O), 1663.97 (C=O), 1527.60 (C=N); ^1^H NMR (300 MHz, DMSO-CDCl_3_): *δ* [ppm], 6.85–7.85 (m, 8H, Ar-H), 10.9 (s, 1H, CONH), 11.6 (s, 1H, indole NH), 14.4 (s, 1H, OH phenolic); MS: *m*/*z* 281 (M^+^).

*(3) 2-Hydroxy-N*′*-(5-methyl-2-oxoindolin-3-ylidene)benzohydrazide (* ***VIIb*** *).* IR (KBr) cm^−1^: 3170 (NH), 3060 (C-H), 1719 (C=O), 1664 (C=O), 1527 (C=N). ^1^H-NMR (DMSO-*d*_6_): *δ* [ppm], 2.6 (s, 3H, CH_3_), 7.2 (s, 1H, Ar-H), 7.4 (d, 1H, Ar-H) 7.6 (t, 2H, Ar-H), 7.7 (d, 1H, Ar-H), 7.92 (d, 2H, Ar-H), 11.6 (s, 1H, indole NH), 11 (s, 1H, CONH), 14.1 (s, 1H, OH phenolic). MS: *m*/*z* 295 (M^+^).

*(4) 2-Hydroxy-N*′*-(5-chloro-2-oxoindolin-3-ylidene)benzohydrazide (* ***VIIc*** *).* IR (KBr) cm^−1^: 3170.58 (NH), 1724.26 (C=O), 1675.74 (C=O), 1528.08 (C=N), 768 (C-Cl). ^1^H-NMR (DMSO-*d*_6_): *δ* [ppm]: 6.92 (s, 1H, Ar-H), 7.21 (d, 1H, Ar-H), 7.52 (t, 2H, Ar-H), 7.81 (d, 1H, Ar-H), 7.94 (d, 2H, Ar-H), 10.4 (s, 1H, CONH), 12.6 (s, 1H, indole), 14.6 (s, 1H, OH phenolic). MS: *m*/*z* 315 (M^+^), 317 (M^+2^).

*(5) 2-Hydroxy-N*′*-(5-bromo-2-oxoindolin-3-ylidene)benzohydrazide (* ***VIId*** *).* IR (KBr) cm^−1^: 3236.95 (NH), 1676.20 (C=O), 1628.43 (C=O), 1542.03 (C=N). ^1^H-NMR (DMSO-*d*_6_): *δ* [ppm]: 7.02 (s, 1H, Ar-H), 7.42 (d, 1H, Ar-H), 7.62 (t, 2H, Ar-H), 7.84 (d, 1H, Ar-H), 7.89 (d, 2H, Ar-H), 11.4 (s, 1H, CONH), 12.2 (s, 1H, indole NH), 14.1 (s, 1H, OH phenolic). MS: *m*/*z* 360 (M^+^).

*(6) 3-(2-(2-Hydroxybenzoyl)hydrazono)-2-oxoindoline-5-carboxylic Acid (* ***VIIe*** *).* IR (KBr) cm^−1^: 3289.98 (NH), 1702.00 (C=O), 1676.94 (C=O), 1529.67 (C=N). ^1^H-NMR (DMSO-*d*_6_): 7.12 (s, 1H, Ar-H), 7.32 (d, 1H, Ar-H), 7.42 (t, 2H, ArH), 7.91 (d, 1H, ArH), 7.94 (d, 2H, ArH), 10.1 (s, 1H, CONH), 12.6 (s, 1H, indole NH), 14.0 (s, 1H, OH phenolic), 14.8 (s, 1H, carboxylic). MS: *m*/*z* 326 (M^+1^).

*(7) 2-Hydroxy-N*′*-(1-methyl-2-oxoindolin-3-ylidene)benzohydrazide (* ***VIIf*** *).* IR (KBr) cm^−1^: 3160 (NH), 3122 (C-H), 1731.90 (C=O), 1665.9 (C=O), 1542.60 (C=N). ^1^H-NMR (DMSO-*d*_6_): *δ* [ppm], 3.8 (s, 3H, CH_3_), 6.95–7.82 (m, 8H, Ar-H), 11.9 (s, 1H, CONH), 13.8 (s, 1H, OH phenolic). MS: *m*/*z* 296 (M^+1^).

*(8) 2-Hydroxy-N*′*-(1-acetyl-2-oxoindolin-3-ylidene)benzohydrazide (* ***VIIg*** *).* IR (KBr) cm^−1^: 3260 (NH), 3068 (C-H), 1791.90 (C=O), 1685.47 (C=O), 1542.60 (C=N). ^1^H NMR (DMSO-*d*_6_): *δ* [ppm], 4.7 (s, 3H, CH_3_), 6.82–7.80 (m, 8H, Ar-H), 11.5 (s, 1H, CONH), 13.4 (s, 1H, OH phenolic). MS: *m*/*z* 323 (M^+^).

*(9) 3-(2-(2-Hydroxybenzoyl)hydrazono)-2-oxoindoline-7-carboxylic Acid (* ***VIIh*** *).* IR (KBr) cm^−1^: 3219.98 (NH), 1722 (C=O), 1677.94 (C=O), 15249.67 (C=N); ^1^H NMR (300 MHz, DMSO-CDCl_3_): 7.02 (d, 1H, Ar-H), 7.22 (d, 1H, Ar-H) 7.4 (t, 2H, Ar-H), 7.87 (t, 1H, ArH), 7.9 (d, 2H, ArH), 11.1 (s, 1H, CONH), 12.0 (s, 1H, indole NH), 14.2 (s, 1H, OH phenolic), 14.5 (s, 1H, carboxylic); MS: *m*/*z* 326 (M^+1^).

*(10) 2-Hydroxy-N*′*-(7-chloro-2-oxoindolin-3-ylidene)benzohydrazide (* ***VIIi*** *).* IR (KBr) cm^−1^: 3170.58 (NH), 1724.26 (C=O), 1675.74 (C=O), 1528.08 (C=N), 762 (C-Cl); ^1^H-NMR (300 MHz, DMSO-CDCl_3_): *δ* [ppm]: 6.92 (d, 1H, ArH), 7.21 (d, 1H, ArH), 7.52 (t, 2H, ArH), 7.81 (t, 1H, ArH), 7.94 (d, 2H, ArH), 10.4 (s, 1H, CONH), 12.6 (s, 1H, indole NH), 14.6 (s, 1H, OH phenolic); MS: *m*/*z* 315 (M^+^), 317 (M^+2^).

*(11) 2-Hydroxy-N*′*-(7-bromo-2-oxoindolin-3-ylidene)benzohydrazide (* ***VIIj*** *).* IR (KBr) cm^−1^: 3236 (NH), 1674 (C=O), 1628 (C=O), 1542, (C=N); ^1^H-NMR (300 MHz, DMSO): *δ* [ppm]: 7.20 (d, 1H, ArH), 7.40 (d, 1H, ArH), 7.60 (t, 2H, ArH), 7.84 (m, 1H, ArH), 7.89 (d, 2H, ArH), 11.4 (s, 1H, CONH), 12.8 (s, 1H, indole NH), 14.12 (s, 1H, OH phenolic); MS: *m*/*z* 345 (M^+1^).

## 4. Biological Activities

### 4.1. Acute Toxicity Studies

The study was conducted in accordance with OECD guidelines (Testing of Chemical Number 423). Healthy and adult male albino Swiss mice were used in this investigation. Animals were fasted for 24 hours and divided into groups of six animals. The test compounds, suspended in normal saline, were administered intraperitoneally, in doses of 10 mg to 1000 mg per kg (b.w.). The control groups of animals received only the vehicle (normal saline). The animals were observed for 48 hours from the time of administration of test compound, in a dose of 1000 mg/kg produce mortality (LD_50_). Soon, tenth of the LD_50_, that is, 100 mg/kg (ED_50_), was selected as a dose for an anti-inflammatory.

### 4.2. Anti-Inflammatory Activity by Carrageenan Induced Rat Hind Paw Edema Method

In carrageenan model [29] albino rats of all groups were treated with subcutaneous injection of 0.1 mL of 1% w/v solution of carrageenan into the subplantar region of the right hind paw. The paw was marked with permanent marker at the planter region where the paw volume was to be measured. The indomethacin 10 mg/kg and test compounds 100 mg/kg were suspended in 0.3% sodium carboxymethyl cellulose. The test compounds and vehicle (control) were administered* i.p.* one hour after the injection of carrageenan in subplantar region of right paw. Mean normal paw volume was measured 30 min prior to carrageenan injection by using plethysmometer. Mean increase in the paw volume for control group (after carrageenan injection) and test group was measured at 1 hr, 2 hr, 3 hr, and 4 hr. Percent inhibition of inflammation after test/standard was calculated using the formula. The mean percent inhibition of indomethacin and tested compounds at 10 mg kg^−1^ concentrations was compared with control using repeated measures ANOVA with Dunnet's test. The data obtained is expressed as mean standard error of mean (SEM):

(1) %  Inhibition=Mean edema of control group − Mean edema of testMean edema of control group×100.

### 4.3. Molecular Docking

**Figure 1:**
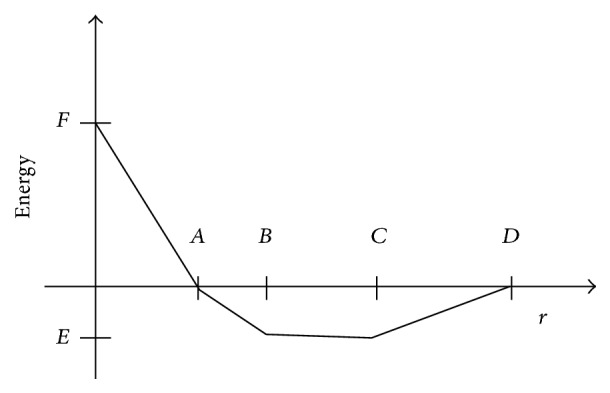
Graphical representation of functional of PLP scoring.

Docking simulation was done by GRIP batch docking method. GRIP docking employs the PLP scoring function in a novel way for fast and accurate capturing of ligand receptor interactions in the active site of proteins. For this purpose, VLife MDS 4.3 (http://www.vlifesciences.com) incorporates the Piecewise Linear Pairwise Potential (PLP) [30, 31] scoring function in GRIP docking method that includes interactions like hydrogen bonding, steric interactions, Vanderwaal's interactions, hydrophobic interactions, and electrostatic interactions. The PLP score is designed to enable flexible docking of ligands to perform a full conformational and positional search within a rigid binding site. The functional form of the ligand-protein interaction energy in PLP scoring function is shown in Figure 1. Parameters of the atomic pairwise ligand–protein potential are as follows: for steric interactions, *A* = 3.4, *B* = 3.6, *C* = 4.5, *D* = 5.5, *E* = −0.4, and *F* = 20.0; for hydrogen bond interactions, *A* = 2.3, *B* = 2.6, *C* = 3.1, *D* = 3.4, *E* = −2.0, and *F* = 20.0. The units of *A*, *B*, *C*, and *D* are Angstrom; for *E* and *F* the units are arbitrary energy units [32].

The X-ray crystal structure of COX-1 and COX-2 (PDB codes: 3N8Y and 3LN1, resp.) was downloaded from the protein data bank protein complexed with indomethacin and celecoxib, respectively. Water molecules were removed and hydrogen was added to the crystal structure of protein, refined by completing the incomplete residues and missing residues. The side chain hydrogen was then added to the crystal structure and its positions were optimized up to the rms gradient 1 by aggregating the other part of the receptor. The optimized receptor was then saved as mol2 file and used for docking simulation. The 2D structure of test compounds built and then converted into the 3D with the help of VLife MDS 4.3 software. The 3D structures were then energetically minimized up to the rms gradient of 0.01 using Merck Molecular Force Field (MMFF). All optimized ligands were docked into active site of COX proteins; the active site with the largest surface area is selected for docking. The parameters fixed for docking simulation were like this number of placements: 30, rotation angle: 30°, exhaustive method, scoring function: PLP score. By rotation angle, the ligand gets rotated for different poses. By placements, the method will check all the 30 possible placements into the active site pocket and results in few best placements out of 30. For each ligand, all the conformers with their best placements and their dock scores will be saved in output folder. The method also highlights the best placement of best conformer of one particular ligand which is having best (minimum) PLP score. After completion of the docking process, the minimum interaction energy between each ligand and COX proteins for the best ligand pose inside the receptor cavity was obtained as the PLP score. The PLP scores were compared with reference compounds.

## 5. Results and Discussion

Some of the new isatin derivatives were obtained by condensation of 2-hydroxybenzohydrazide with various isatins in presence of glacial acetic acid depicted in Scheme 2. Physical data of all synthesized compounds are shown in Table 1 [Fig sch1].

### 5.1. *In Vivo* Anti-Inflammatory Activity

All synthesized compounds were evaluated for* in vivo* anti-inflammatory activity at the dose of 100 mg/kg by carrageenan induced paw edema method. From the data it was revealed that all tested compounds significantly reduced carrageenan induced edema and the results were presented in Table 2. Among the tested compounds,** VIIc** (R_1_ = 5-Cl) and** VIId** (R_1_ = 5-Br) may be considered as potent anti-inflammatory agents and comparable with standard anti-inflammatory drug, indomethacin. Interestingly, derivatives with halo substitution at position C-5 and position C-7 of the isatin, compounds** VIIc** (R_1_ = 5-Cl),** VIId** (R_1_ = 5-Br),** VIIi** (R_1_ = 7-Cl), and** VIIj** (R_1_= 7-Br) exhibited substantial activity with 65%, 63%, 62%, and 60% edema reduction. On the other hand, compounds with alkyl and acetyl substitution at position N-1 of the isatin and compounds** VIIf** (R_2_ = CH_3_),** VIIg** (R_2_ = COCH_3_) are next in the order of activity with 57.58% of edema reduction, whereas compounds** VIIb** (R_1_ = 5-CH_3_),** VIIa** (R_1_ = R_2_ = H),** VIIe**, and** VIIh** (5&7 COOH) exhibited 52%, 54%, 55%, and 49% of oedema reduction, respectively.

Compounds having substitution with electron withdrawing groups at C-5 and C-7 position of isatin moieties showed more anti-inflammatory activity whereas the other substitutions on various positions of isatin nucleus are next in the order of activity.

### 5.2. Molecular Docking Studies

**Figure 2:**
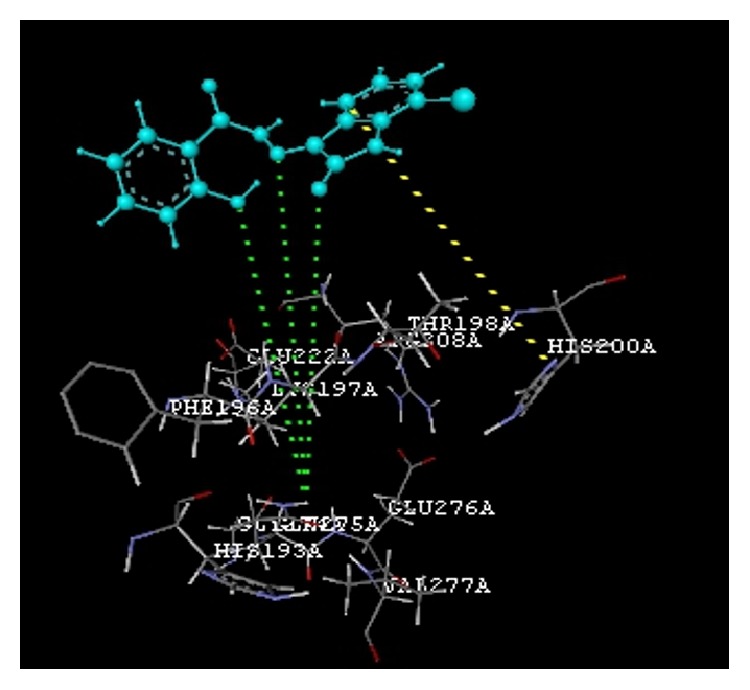
Compound** VIIc** (R = 5-Cl) interactions.

**Figure 3 fig3:**
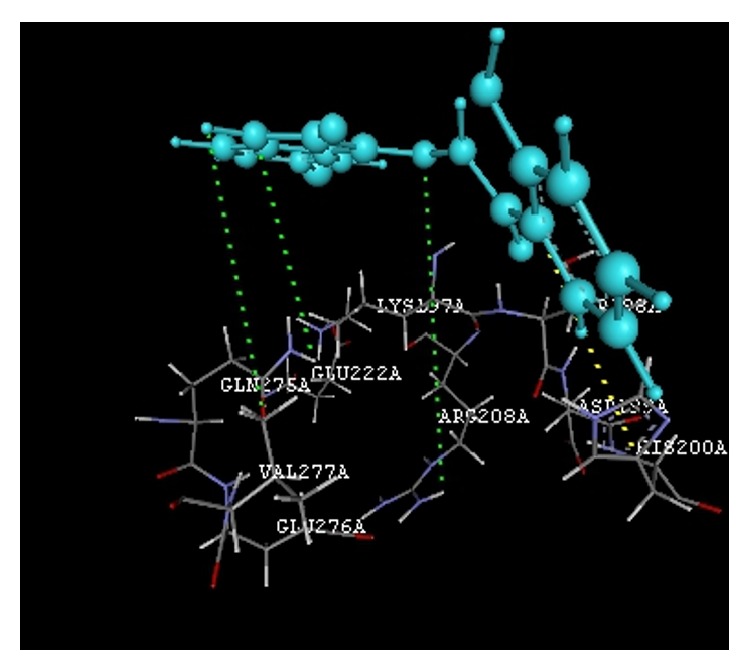
Compound** VIId** (R = 5-Br) interactions.

**Figure 4 fig4:**
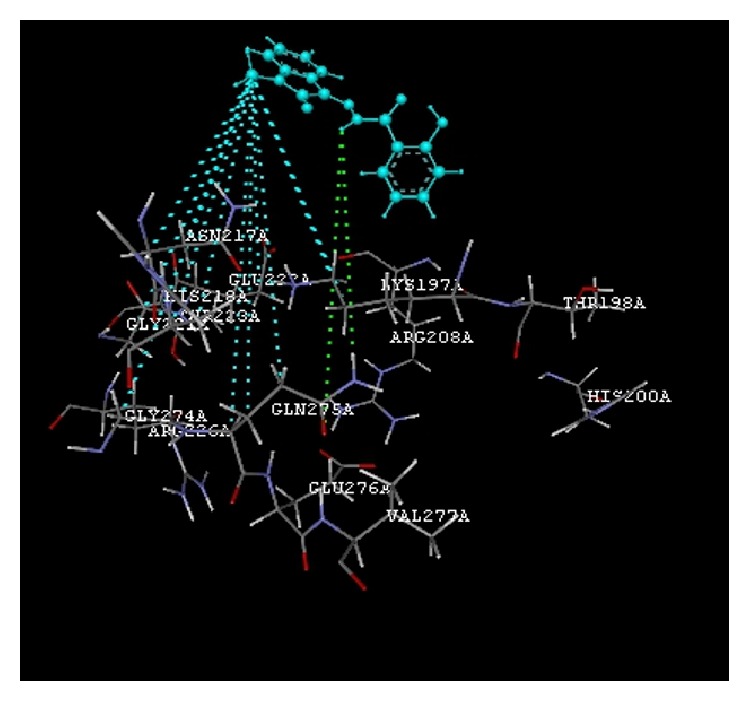
Compound** VIIf** (R = 1-CH_3_) interactions.

**Figure 5 fig5:**
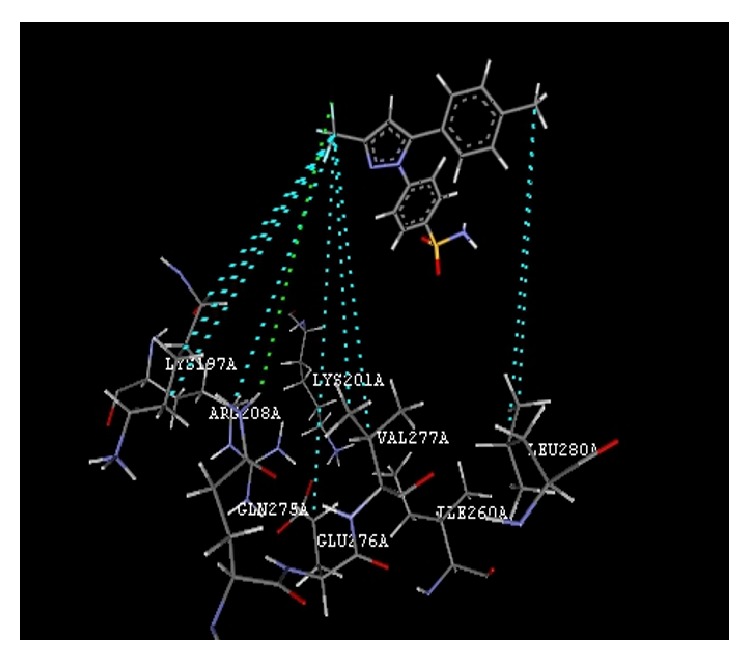
Celecoxib interactions.

**Figure 6 fig6:**
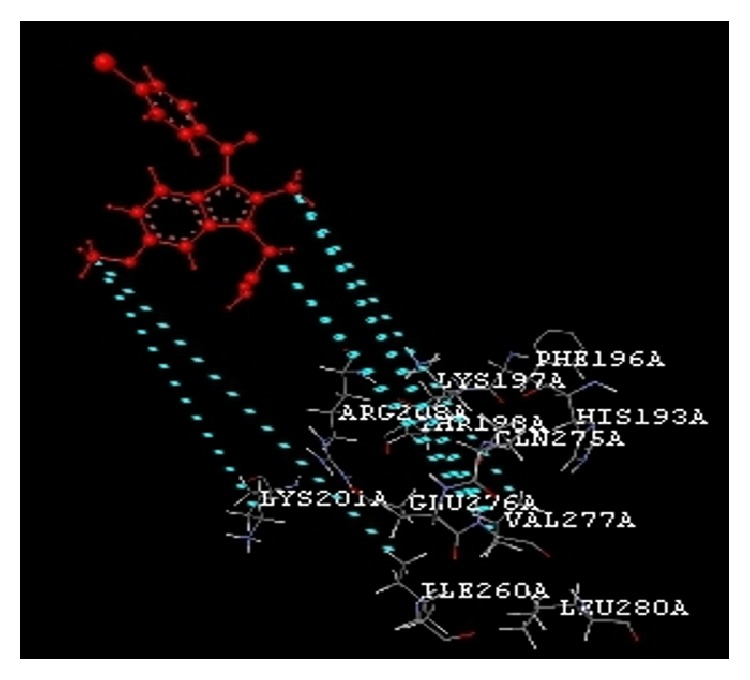
Indomethacin interactions.

**Figure 7:**
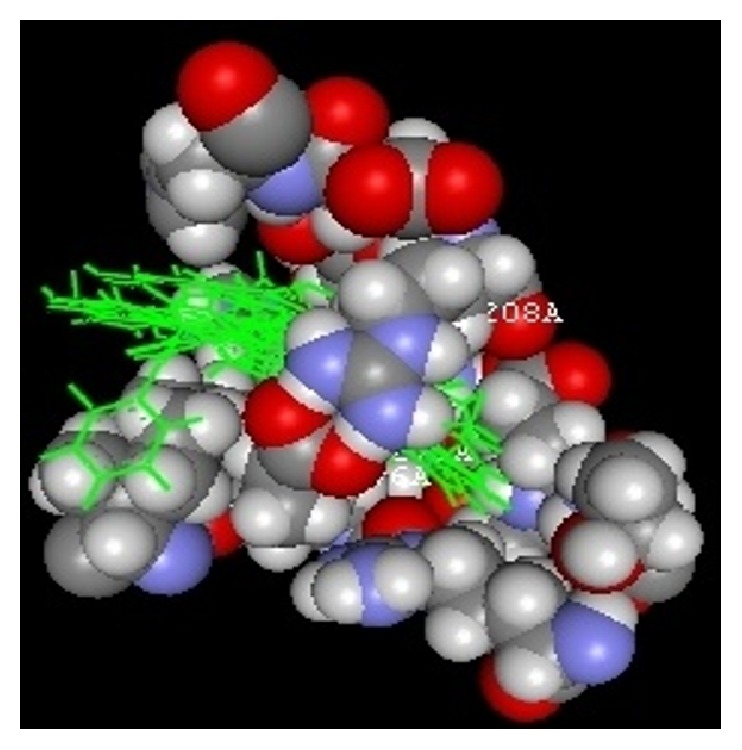
All docked complex.

The docking study was performed using the VLife MDS 4.3. All the ten 2-hydroxy-N^1^-(2-oxo-1,2-dihydro-3H-indol-3-ylidene)benzohydrazide derivatives were docked into the active site of the enzymes COX-1 (PDB ID: 3N8Y) and COX-2 (PDB ID: 3LN1) which showed better docking scores than the reference compounds indomethacin and celecoxib and the results were presented in Table 3. Compounds** VIIe** and** VIIh** showed good docking scores −68.73, −43.30 with COX-2 enzyme even though they were not considered good anti-inflammatory agents because they are also having good dock scores −26.79, −27.28 with COX-1 enzymes so these may be the causes of gastric irritation. The compounds** VIIc**,** VIId**, and** VIIf** are considered as good anti-inflammatory agents because of good binding scores −57.27, −62.02, and −58.18 into the active site of COX-2 and least dock scores −8.03, −9.17, and −8.94 on COX-1 enzymes. Compounds** VIIc** and** VIId** showed hydrogen bond interaction with Gln-275 and aromatic interaction with His-193 and compound** VIIf** showed hydrophobic interactions with Lys197, Asn217, His218, Gly221, Gly274, Glu222, and Gln275 with COX-2 enzyme whereas the reference compounds showed hydrophobic, van der waals interactions presented in Table 4, Figures 2 [Fig fig3] [Fig fig4] [Fig fig5] [Fig fig6]–7.

## 6. Conclusions

We described herein the synthesis and characterization of a novel 2-hydroxy-N^1^-(2-oxo-1,2-dihydro-3H-indol-3-ylidene)benzohydrazide derivatives. All the synthesized compounds are subjected for the evaluation of* in vivo* anti-inflammatory activity at dose 100 mg/kg. Among all, the compounds** VIIc** and** VIId** had good anti-inflammatory activity. Electron withdrawing groups seemed to be necessary factors in providing higher anti-inflammatory activity.

The molecular docking studies further help in understanding the various interactions between the ligands and enzyme active sites in detail and thereby help to design novel potent inhibitor. The docking experiments were carried out for all the synthesized compounds on COX-1 and COX-2 enzymes and compared the docking score with reference compounds indomethacin and celecoxib. The compounds** VIIc**,** VIId**, and** VIIf** showed higher binding score, which are further attributed to the* in vivo* activity of these compounds.

## Figures and Tables

**Scheme 1 sch1:**
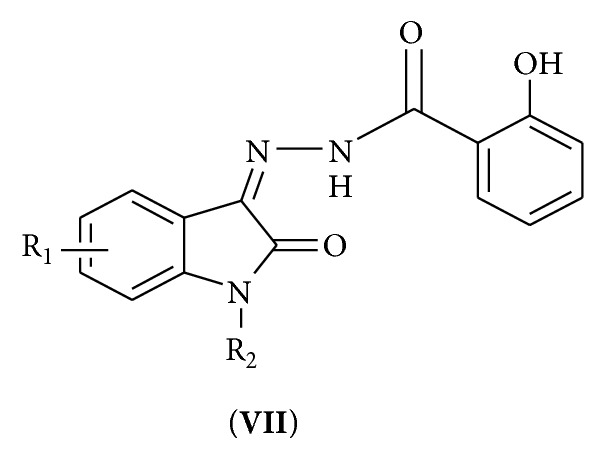


**Table 1:** Physical data of the newly synthesized compounds (**VIIa**–**j**). See Scheme 1.

S. number	Compounds	R_1_	R_2_	Mol. F	Mol. wt.	M.P (°C)	% yield
1	**VIIa**	H	H	C_15_H_11_N_3_O_3_	281	290–292	88
2	**VIIb**	5-CH_3_	H	C_16_H_13_N_3_O_3_	295	285–287	84
3	**VIIc**	5-Cl	H	C_15_H_10_N_3_O_3_Cl	315	294–296	82
4	**VIId**	5-Br	H	C_15_H_10_N_3_O_3_Br	360	302–306	86
5	**VIIe**	5-COOH	H	C_16_H_11_N_3_O_5_	325	286–288	84
6	**VIIf**	H	N-CH_3_	C_16_H_13_N_3_O_3_	295	180–182	82
7	**VIIg**	H	N-COCH_3_	C_17_H_13_N_3_O_4_	323	152–155	72
8	**VIIh**	7-COOH	H	C_16_H_11_N_3_O_5_	325	287–289	82
9	**VIIi**	7-Cl	H	C_15_H_10_N_3_O_3_Cl	315	295–297	78
10	**VIIj**	7-Br	H	C_15_H_10_N_3_O_3_Br	360	300–302	75

**Table 2:** Showing the mean paw edema volume (*n* = 6) for newly synthesized compounds (100 mg/kg bw) compared to standard drug indomethacin (10 mg/kg bw).

S. number	Compound (100 mg/kg)	1 hr	2 hr	3 hr	4 hr
1	**Control**	0.75 ± 0.05	0.82 ± 0.02	0.94 ± 0.09	1.2 ± 0.1
2	**VIIa**	0.65 ± 0.01	0.62 ± 0.05	0.58 ± 0.03	0.55 ± 0.01 (54)^a^
3	**VIIb**	0.64 ± 0.08	0.61 ± 0.05	0.60 ± 0.01	0.57 ± 0.02 (52)^a^
4	**VIIc**	0.55 ± 0.02	0.52 ± 0.04	0.48 ± 0.07	0.42 ± 0.01 (65)^b^
5	**VIId**	0.58 ± 0.03	0.56 ± 0.09	0.50 ± 0.04	0.44 ± 0.08 (63)^b^
6	**VIIe**	0.63 ± 0.05	0.61 ± 0.02	0.58 ± 0.04	0.54 ± 0.01 (55)^b^
7	**VIIf**	0.60 ± 0.05	0.57 ± 0.08	0.55 ± 0.04	0.51 ± 0.03 (57)^b^
8	**VIIg**	0.59 ± 0.01	0.56 ± 0.07	0.54 ± 0.03	0.50 ± 0.05 (58)^b^
9	**VIIh**	0.69 ± 0.06	0.67 ± 0.02	0.64 ± 0.04	0.61 ± 0.01 (49)^a^
10	**VIIi**	0.54 ± 0.03	0.52 ± 0.01	0.50 ± 0.08	0.45 ± 0.06 (62)^b^
11	**VIIj**	0.52 ± 0.04	0.50 ± 0.08	0.46 ± 0.01	0.48 ± 0.02 (60)^b^
12	Indomethacin (10 mg/kg)	0.51 ± 0.02	0.47 ± 0.09	0.42 ± 0.04	0.38 ± 0.01 (68)^b^

All the values are expressed as mean ± SD (*n* = 6); ^a^ *p* < 0.01, ^b^ *p* < 0.001 versus control.

Results in parenthesis are % of mean inhibition.

**Table 3:** Docking scores (PLP Scores) of comparative COX-1 and COX-2 enzymes.

S. number	Compound	COX-1	COX-2
1	**VIIa**	−10	−65.52
2	**VIIb**	−8.72	−48.97
3	**VIIc**	−8.03	−57.27
4	**VIId**	−9.17	−62.02
5	**VIIe**	−26.79	−68.73
6	**VIIf**	−8.94	−58.18
7	**VIIg**	−11	−38.42
8	**VIIh**	−27.28	−43.3
9	**VIIi**	−10.51	−45.45
10	**VIIj**	−14.45	−51.83
11	Indomethacin	−18.91	−57.37
12	Celecoxib	−8.05	−65.05

**Table 4:** Binding interactions of cyclooxygenase enzyme.

Compounds	COX-1	COX-2	Hydrogen bonds		Aromatic bonds		Hydrophobic bonds	
			Dist.	Amino acid	Dist.	Amino acid	Dist.	Amino acid
**VIIc**	−8.03	−57.27	2.073	Gln275	4.702	His193		
			2.354					

**VIId**	−9.17	−62.02	2.073	Gln275	5.211	His193		
			2.496					

**VIIf**	−8.94	−58.18	2.378	Gln275	4.198	His193	4.473	Lys197
			2.51				3.975	Asn217
							3.295	His218
							4.336	Gly221
							4.675	Glu222
							3.663	Gln275

Indomethacin	−18.91	−57.37					4.865	Lys201
							4.396	Ile260
							3.802	Val277

Celecoxib	−10.8	65.05					4.356	Leu280
							4.909	Val277
							4.953	Glu276
							4.986	Arg208
							4.589	Lys197
							2.305	Gln275
